# Dopamine transporter 3'UTR VNTR genotype is a marker of performance on executive function tasks in children with ADHD

**DOI:** 10.1186/1471-244X-8-45

**Published:** 2008-06-17

**Authors:** Sherif Karama, Natalie Grizenko, Edmund Sonuga-Barke, Alysa Doyle, Joseph Biederman, Valentin Mbekou, Anna Polotskaia, Marina Ter-Stepanian, Rosherrie De Guzman, Johanne Bellingham, Sarojini Sengupta, Ridha Joober

**Affiliations:** 1 Department of Psychiatry, McGill University, Montreal, Canada; 2 Department of Psychiatry, Douglas Mental Health University Institute, Montreal, Canada; 3 Department of Psychiatry, Neurology and Neurosurgery, McGill University, Montreal, Canada; 4 Department of Human Genetics, McGill University, Montreal, Canada; 5 School of Psychology, Southampton University, Southampton, UK; 6 Department of psychiatry, Harvard Medical School, Boston, USA; 7 Douglas Hospital Research Centre, 6875 LaSalle blvd., Verdun, QC, H4H 1R3, Canada

## Abstract

**Background:**

Attention-Deficit/Hyperactivity Disorder (ADHD) is a heterogeneous disorder from both clinical and pathogenic viewpoints. Executive function deficits are considered among the most important pathogenic pathways leading to ADHD and may index part of the heterogeneity in this disorder.

**Methods:**

To investigate the relationship between the dopamine transporter gene (*SLC6A3*) 3'-UTR VNTR genotypes and executive function in children with ADHD, 196 children diagnosed with ADHD were sequentially recruited, genotyped, and tested using a battery of three neuropsychological tests aimed at assessing the different aspects of executive functioning.

**Results:**

Taking into account a correction for multiple comparisons, the main finding of this study is a significant genotype effect on performances on the Tower of London (F = 6.902, p = 0.009) and on the Wechsler Intelligence Scale for Children, Third Edition (WISC-III) Freedom From Distractibility Index (F = 7.125, p = 0.008), as well as strong trends on Self Ordered Pointing Task error scores (F = 4,996 p = 0.026) and WISC-III Digit Span performance (F = 6.28, p = 0.023). Children with the 9/10 genotype exhibited, on average, a poorer performance on all four measures compared to children with the 10/10 genotype. No effect of genotype on Wisconsin Card Sorting Test measures of performance was detected.

**Conclusion:**

Results are compatible with the view that *SLC6A3 *genotype may modulate components of executive function performance in children with ADHD.

## Background

ADHD is a heterogeneous disorder characterized by age-inappropriate inattention, motor activity, and/or impulse control. Its high prevalence in school-aged children (8 to 12%) [1] in combination with its significant negative impact on school and social adjustment as well as family wellbeing make it one of the most important public health problems in the child population. Multiple risk factors interact to increase liability to ADHD [2]. While environmental risk factors such as pregnancy complications [3], family dysfunction [4] and drug abuse during pregnancy [5] have all been implicated in the etiology of ADHD, there is overwhelming evidence from twin studies that genes also contribute to the development of ADHD [2,6,7].

There has been a strong focus on the dopamine transporter in ADHD as psychostimulants, the first line medication for ADHD, have been shown to block this transporter as part of their mechanism of action [8]. Further, single photon emission computed tomography studies show high rates of striatal dopamine transporter activity in drug naive adults with ADHD [9-11]. In keeping with this, a pooled odds ratio (OR) for ADHD from family studies for the dopamine transporter (DAT1) gene 10 repeat allele was found to be significant [6] and other meta-analyses have invariably shown positive ORs for this allele [12-16], although not necessarily significant ones.

While the above-mentioned genetic studies have examined allelic associations between specific polymorphisms and ADHD, genotypic approaches have also yielded support for DAT1 involvement. For instance, Loo et al. have reported that in patients with ADHD, there is an association between 10/10 DAT1 genotype and increased errors on a vigilance task when compared to a compound group composed of children having the 9/9 or 9/10 genotypes [17]. Along similar lines, children with ADHD and carriers of both the 10/10 DAT1 genotype as well as a 7-repeat DRD4 allele have been reported as having a lower IQ than a combined group of carriers of other genotypes [18]. Further, poor response to methylphenidate was associated, in ADHD children, with DAT1 10-repeat allele homozygosity in both prospective and naturalistic studies [19-21].

Due to the relatively small frequency of the 9/9 genotype in the general population and to the consequently small sample sizes generally acquired for this genotype, 10/10 genotype groups are usually compared to a combined group of subjects with the 9/9 and 9/10 genotypes (as well as other very rare genotypes). This being said, a few recent studies have compared genotypes separately. For instance, Cornish et al. [22] have shown, in normal boys, an association between the 10/10 repeat allele genotype and poorer mean scores on measures of selective attention and response inhibition compared to a group of 9/10 carriers. Joober et al. [23], using a 2-week prospective within subject crossover design have shown that children with ADHD and carriers of the 9/9 genotype displayed a significantly weaker response to methylphenidate than carries of the 9/10 genotype or carriers of the 10/10 genotype. Further, a recent study [24] demonstrated that individuals with ADHD and carriers of the 9/10 genotype tended to fair worse than carriers of the 10/10 genotype on a large set of variables including behavioral problems in childhood and adolescence, mother-teen relations at adolescence, and class rankings in high school. However, no significant differences between genotype groups were found in Wisconsin Card Sorting Test performance. The majority of findings in this study essentially supports the view that young carriers of the 9/10 genotype tend to fair worse than their 10/10 counterparts from a psychopathological perspective. While this work appears to contradict above-mentioned studies demonstrating a disadvantage for carriers of the 10/10 genotype, it is noteworthy that it's the first study, to the best of our knowledge, to separately look at DAT1 gene genotype in relation to behavior in children with ADHD.

Overall, these patterns of results are consistent with a high degree of heterogeneity in genetic effects and with the view that current candidate genes are only indirectly responsible for ADHD as they may instead be associated with specific clinical variants and/or behavioral dimensions of ADHD yet to be precisely identified. If this is the case, selecting candidate genes on the basis of their potential implication in modulating intermediate phenotypes (or endophenotypes) relevant to ADHD could improve the robustness of genetic results. While endophenotypes can exist at a number of levels, most studies to date have focused on the neuropsychological level. One such pathway with considerable empirical support implicates dysregulation of executive function processes (defined as the neurocognitive processes that maintain an appropriate problem-solving set to attain a future goal [25,26]). Factor analyses of batteries of measures of executive function have revealed the four following factors: 1) response inhibition and execution, 2) working memory and updating, 3) set-shifting and task-switching, and 4) interference control [26-30]. Although most mental tasks involve almost every component of executive functioning to some degree, some tasks tap relatively highly into specific factors.

While traditionally, executive function tasks have been believed to rely mostly on the prefrontal cortex, the involvement of the basal ganglia is now being recognized [31,32]. For instance, it has been shown that fronto-striatal pathway disconnection disrupts normal performance on classical cognitive frontal tasks in rats [33] and that the executive dysfunction frequently observed in Parkinson's disease is associated with altered pallidal-frontal processing [34]. In keeping with this, one of the most replicated morphological alterations in brains of children with ADHD is small caudate and pallidum volumes [35].

Finding an association between executive function dysregulation and ADHD together with demonstration that dopamine neurotransmission is critical for normal executive functioning would highlight the plausibility of executive function as an endophenotype in ADHD. From a genetic standpoint, the DAT1 gene is known to be preferentially expressed in the basal ganglia and has been reported to influence caudate volume [36] and aspects of executive functioning in normal subjects [22]. Furthermore and in keeping with the above, the dopamine transporter has been argued to play a critical role in regulating cortical signal-to-noise ratio during working memory via a cortico-striato-thalamo-cortical pathway [37]. As reviewed by Doyle et al[2], family, twin, and adoption studies all suggest that EFs may index the familial/genetic liability to ADHD. Given the data discussed above, DAT1 gene is a compelling candidate gene to examine in relation to both EF performance and ADHD.

The present study examined executive function performance in children with ADHD in relation to the Variable Number of Tandem Repeats (VNTR) located in the 3' untranslated (UTR) region of the DAT1 gene in order to further characterize the association between genotype and behavior in children with ADHD. It is hypothesized that DAT1 gene genotype has an effect on executive function performance.

## Methods

### Subjects

Children were sequentially recruited from the Disruptive Behavior Disorders Program and the Children Outpatient Clinics of the Douglas Hospital. They were referred to these specialized care facilities by school teachers, community social workers and pediatricians.

To be included in this study, children were required to be between 6 and 12 years of age and meet DSM-IV diagnosis criteria for ADHD [38]. Diagnosis was based on a structured clinical interview of parents using the fourth edition of the diagnostic interview schedule for children-parental report (DISC-IV) [39], school reports, teachers' reports, and clinical observation of the subject. In the majority of cases, mothers were the primary informants for the collection of clinical information. The Child Behavioral Checklist [40], a scale that assesses several behavioral domains was completed by parents for each child. Children having a history of mental retardation, with an IQ ≤ 70 as measured by the Wechsler Intelligence Scale for Children-third edition (WISC-III) [41], a history of Tourette syndrome, pervasive developmental disorder, psychosis, bipolar disorder or any medical condition or impairment that would interfere with the ability of the child to complete the study, were excluded. All diagnostic and baseline evaluations were conducted at the Douglas Hospital.

### Psychometric assessment

In order to capture the gist of the four factors of executive functioning reported above (*ie *1- response inhibition and execution (includes planning), 2- working memory and updating, 3- set-shifting and task-switching, and 4- interference control), our neuropsychological test battery comprised the Wisconsin Card Sorting Test (WCST) [42], the Self Ordered Pointing Task (SOPT) [43], the Tower of London (TOL), which is a variant on the Tower of Hanoi task [44], and the WISC-III from which a Freedom from Distractibility Index (FFDI) was extracted.

The WCST, the SOPT, and the TOL were administered by trained research personnel as described in Taerk et al. [45]. For both tests, all children were assessed while not having taken medication for at least one week. Both research personnel and subjects were blinded to the genotype status of the subjects.

Furthermore, the WISC-III was administered by a trained psychologist to all children not having had it administered in the previous year at school. Where the WISC-III had been administered in the previous year, records of the evaluation were made available by the school authorities. In all cases where records were complete, the Freedom from Distractibility Index (FFDI) was derived from the Arithmetic and Digit Span subtest scores. 17% of children (n = 16) with the 9/10 genotype and 14% of children (n = 18) with the 10/10 genotype were tested at school.

In the WCST, the child is asked to sort cards according to three different criteria (color, number, or shape of the symbols present on the cards). Feedback on whether the child achieves a correct or incorrect match is given after each trial. The matching criterion changes after ten consecutive correct matches and the child has to identify the new matching criterion using the feedback (correct/incorrect) provided to him. While the WCST is a task where one has to hold active information in working memory in order to identify the matching criterion, it arguably taps mostly into response inhibition capacity and into set-shifting and task switching ability. Evidence for the high reliability and validity of the WCST for various normal and clinical populations has been reported in many studies [42]. While the WCST perseverative errors score has frequently been examined in previous work pertaining to ADHD [46], non-perseverative errors, total errors, number of trials to complete the first category, as well as number of categories completed were also examined here.

In the SOPT, series of matrices of 6, 8, 10 and 12 images are presented to the child. The child is asked to chose, by pointing, one different image on each page. Errors occur when the child points to images that he pointed to in previous presentations of the set of images. Each set is presented to the child three times. While performing the SOPT, the demand on keeping information active in working memory is very high. SOPT scores have been shown to highly correlate with measures of working memory, strategy utilization and planning (*ie *response inhibition and execution), but not with measures of interference control [47]. Test-retest reliability for the SOPT total errors score has been shown to be high while stability of other SOPT indices, including perseverative errors, has been shown to be rather poor [47]. As children with ADHD have been reported to have a poorer performance on the SOPT total errors score than normal controls [48,49], this index has been examined here.

In the TOL, the child is presented with colored beads with a hole in them. These beads are placed on three pegs in a given initial pattern. The child is asked to move the beads, one by one, in order to match a target pattern. Beads can only be moved from peg to peg *ie *they cannot be kept in one's hand. Importantly, subjects are told to plan ahead and try to match the target pattern with the least possible number of moves. The TOL has twelve items of increasing difficulty. The TOL taps relatively highly into working memory as well as into strategy utilization and planning [50]. The standard scores for each of the 12 items were examined.

Numerous factor analyses of the WISC-III have revealed a Freedom from Distractibility Index (FFDI) constituted by the Arithmetic and Digit Span subtests [41]. Performance on the FFDI is very likely highly influenced by deficits in interference control and working memory and has been found to be relatively low for children with ADHD [41]. While in the Arithmetic task, children are asked to solve arithmetic problems, in the Digit Span subtest of the WISC-III, children are required to repeat verbatim or in reverse order, strings of digits recited at the rate of one per second. The length of the strings is gradually increased. The test is ended when the child is no longer able to correctly repeat the strings.

The research protocol was approved by the Research Ethics Board of the Douglas Hospital (Montreal, Quebec). Parents were explained the study and provided written consent. Children were explained the study and gave their assent to participate.

### Molecular genetics

Blood samples were collected from affected children and their parents in order to extract DNA for the purpose of genetic analyses. The VNTR polymorphism in the *SLC6A3 *gene was genotyped using a PCR-based method as previously described [51]. The PCR was performed in a 15 μl total reaction volume containing 1× PCR buffer, 200 uM dNTPs, 100 ng of primers (5'-TGTGGTGTAGGGAACGGCCTGAG-3', 5'-CTTCCTGGAGGTCACGGCTCAAGG-3'), 1 Unit of Taq DNA polymerase and 100 ng of genomic DNA. PCR products were electrophoresed on agarose-TAE gel along with 1 kb and 100 bp DNA ladders, visualized under UV-light and coded according to the length of the PCR product. Genotypes were called by two independent and experienced technicians who were blind to all the clinical data. No discordance in any of the readings was noted.

### Statistical analyses

The VNTR polymorphism has two main alleles: 9- and 10-repeat alleles. Three very rare alleles (3, 7, 8 and 11-repeat) were also identified in 13 subjects (four with the 3/10 genotype, one with the 7/10 genotype, one with the 8/10 genotype, one with the 9/11 genotype, and six with the 10/11 genotype). Because of the very small sample sizes of these latter genotype groups and because only 21 carriers of the 9/9 genotype were detected, we limited our analyses to the groups of children with the 9/10 or 10/10 genotypes. The total sample size of subjects having been genotyped for the DAT1 gene was 251. Of these, 96 had the 9/10 genotype and 127 had the 10/10 genotype.

To test for the effect of genotype on SOPT performance, we implemented a general linear model repeated measures analysis with genotype (2 modalities: 9/10 and 10/10 genotypes) as the between subject factor and the 4 SOPT difficulty level error scores as the within-subject repeated measures. A similar procedure was implemented for the 12 difficulty level scores of the TOL. An analysis of variance was implemented for the WCST and WISC-III scores. Age was used as a covariate except when test scores were already age-standardized (eg WISC-III subtests).

The mean of the absolute values of the correlations between scores on the different outcome variables (WCST, SOPT, TOL, and WISC-III performances) was modest to moderate (mean r = 0.357). Between different tests (eg WCST and WISC subtests), the absolute value of the correlations ranged between a low of 0.042 and a high of 0.379. However, between the different indices of performance on a given test (eg comparing WCST Total errors and WCST Perseverative errors), correlations ranged between 0.495 and 0.874. High correlations between performance values of a given test are expected in cases when performance on one index is in part derived from performance on another index. Taking these correlations into account [52], a Bonferroni correction for multiple comparisons produced a p < 0.011 level of significance as the appropriate cutoff necessary to achieve an overall p < 0.05 alpha level.

## Results

The 10- and 9-repeat allele frequencies were 72% and 28% respectively for the complete sample (ie this calculation includes subjects with the 9/9 genotype). The genotype distribution did not differ from the Hardy-Weinberg equilibrium (χ^2 ^= 0.043, df = 1, p = 0.84). Gender, ethnic group, age, average household income, and severity of behavioral problems as assessed by the CBCL (total, attention, internalizing and externalizing scores, and other behavioral dimensions) were not significantly different between the three genotype groups. Further, no significant differences between genotype groups were found in IQ, in diagnostic subtype of ADHD (inattentive, hyperactive/impulsive and combined) or in the number of ADHD items as assessed by the DISC-IV. In keeping with most studies on ADHD, the sample was characterized by a high prevalence of comorbidity including oppositional defiant disorder and conduct disorder. However, there were no significant differences in comorbid disorders between the genotype groups (see Table 1).

**Table 1:** Demographic and clinical characteristics of sample partitioned by DAT1 genotype

	9/10 genotype	10/10 genotype	Statistics and p values
	n = 96	n = 127	Total n = 223

Age (yrs)	8.8 ± 1.8	9.2 ± 1.9	F = 2.78, p = .10
Proportion of males	80.2% (n = 77)	79% (n = 100)	χ^2 ^= 0.072, p = .79
Proportion of Caucasians	87.5% (n = 84)	82.7% (n = 105)	χ^2 ^= 0.98, p = .32
WISC-III FSIQ	96.7 ± 14.6	99.2 ± 14.4	F = 1.61, p = .21
CBCL total	70 ± 8.1	69.2 ± 9.3	F = 0.41, p = .35
CBCL Attention problems	70.7 ± 9.5	70.8 ± 10.2	F = 0.009, p = .92
CBCL externalisation	70.4 ± 9.4	69.1 ± 10.2	F = 0.65, p = .42
CBCL internalisation	63.7 ± 9.6	64.1 ± 11.4	F = 0.063, p = .80
Proportion with I/H/C ADHD subtype as assessed by the treating psychiatrist	18% (n = 17)/6% (n = 6)/74% (n = 71) 2 missing data points	27% (n = 34)/4% (n = 5)/65% (n = 82) 6 missing data points	χ^2 ^= 3.21, p = .20
DISC-IV Inattentive	6.9 ± 2.2	7.2 ± 2.0	F = 0.51, p = .48
DISC-IV Hyperactive/Impulsive	6.3 ± 2.4	5.6 ± 2.5	F = 3.56, p = .061
with CD/without CD	32% (n = 30)/68% (n = 64) 2 missing data points	24% (n = 30)/76% (n = 94) 3 missing data points	χ^2 ^= 1.6, p = .21
with ODD/without ODD	40% (n = 38)/60% (n = 56) 2 missing data points	41% (n = 52)/59% (n = 74) 1 missing data point	χ^2 ^= 0.016, p = .90
Never/previously medicated	52% (n = 46)/48% (n = 43) 7 missing data points	52% (n = 60)/48% (n = 56) 11 missing data points	χ^2 ^< 0.001, p = .99

Values are percentages or means ± their respective Standard Deviations (SD). Demographic and clinical characteristics were compared between the two groups using the appropriate statistic. WISC = Wechsler Intelligence Scale for Children, 3rd edition; CBCL = Child Behavioural Checklist; I/H/C = Inattentive/Hyperactive/Combined; DISC = Diagnostic Interview Schedule for Children, 4^th ^edition; ODD = Oppositional Defiant Disorder; CD = Conduct Disorder; Percentages are rounded to the nearest unit. Note that in the DSM-IV, a diagnosis of CD supersedes a diagnosis of ODD. However, in order to make the table as informative as possible, this criterion was relaxed. Age was used as a covariate for all F statistics.

The main finding of this study is a significant genotype effect on TOL (F = 6.902, p = 0.009) and FFDI performances (F = 7.125, p = 0.008) as well as a strong trend on SOPT error scores (F = 4,996 p = 0.026) and Digit Span performance (F = 6.28, p = 0.023). Children with the 9/10 genotype had poorer performance on all four measures compared to children with the 10/10 genotype (see Table 2). Scatter plots were examined and these results could not be explained by the presence of outliers in any of the groups (scatter plots not shown). No effect of genotype on WCST measures of performance was detected.

**Table 2:** Comparison of neuropsychological test performance scores for each genotype group

	9/10 genotype	10/10 genotype	F statistic and p value
**Wisconsin Card Sorting Test**			
Perseverative errors raw score	45 ± 22.5	40.7 ± 19.6	F = 1.29; p = 0.26
Non perseverative errors raw score	20.4 ± 12.6	19.1 ± 12.6	F = 0.11; p = 0.74
Total errors raw score	24.7 ± 15.4	22.4 ± 14.2	F = 0.63; p = 0.43
Trials to complete first category	23.9 ± 22.2	21.9 ± 18.5	F = 0.32; p = 0.57
Number of categories completed	4.1 ± 1.7	4.6 ± 1.5	F = 3.17; p = 0.076
**WISC-III**			
Arithmetic	8.5 ± 3.0	9.4 ± 2.6	F = 3.51; p = 0.063
Digit span	7.6 ± 2.3	8.6 ± 2.3	F = 6.28; p = 0.013 *
FFDI	8.0 ± 2.3	9.0 ± 2.0	F = 7.13; p = 0.008 **
**SOPT (4 levels of difficulty)**			
Level 1 number of errors	2.3 ± 1.6	1.9 ± 1.4	
Level 2 number of errors	3.7 ± 2.1	3.0 ± 2.0	
Level 3 number of errors	4.6 ± 2.6	3.7 ± 2.3	
Level 4 number of errors	5.9 ± 3.6	5.0 ± 3.0	
Repeated measures statistic for the SOPT			F = 5.00; p = 0.026 *
**TOL (12 levels of difficulty)**			
Level 1 score	8.2 ± 0.9	8.3 ± 0.9	
Level 2 score	8.0 ± 1.5	8.3 ± 1.1	
Level 3 score	7.6 ± 1.3	7.7 ± 1.1	
Level 4 score	6.1 ± 2.6	6.8 ± 1.9	
Level 5 score	6.5 ± 1.6	6.8 ± 1.4	
Level 6 score	4.3 ± 3.0	4.8 ± 2.7	
Level 7 score	4.5 ± 2.9	4.7 ± 3.0	
Level 8 score	5.2 ± 2.7	5.2 ± 2.6	
Level 9 score	6.3 ± 1.8	6.4 ± 1.7	
Level 10 score	2.8 ± 2.8	3.0 ± 2.8	
Level 11 score	4.0 ± 2.9	4.8 ± 2.4	
Level 12 score	4.4 ± 2.7	4.8 ± 2.5	
Repeated measures statistic for the TOL			F = 6.90; p = 0.009 **

Values are mean ± SD. Statistical analyses for the SOPT and TOL were implemented using a repeated measures design. For this reason, only an overall F statistic is available for each of these two tests.

* → Statistical trend (0.011 < p value < 0.05).

** → Statistically significant (p value < 0.011).

## Discussion

The most important finding of this study is the presence of a significant effect of the *SLC6A3 *3'UTR VNTR genotype on measures of executive function performance in children with ADHD. Indeed, with the exception of the WCST, it was observed that carriers of the 10/10 DAT1 gene genotype had a better performance on various tasks of executive function than carriers of the 9/10 genotype. While differences in results between WCST and the other tests are probably due to multiple complex reasons, it could be tentatively hypothesized that these are due to the fact that the TOL, the SOPT, and the FFDI subtests all exert rather high demands on working memory while the WCST taps mostly on the set-shifting and task-switching factor. Indeed, although the WCST does tap into working memory, its demands on it are relatively low as the child has to keep in mind a maximum of only two out of three possible parameters at a time (ie color, shape, or number). In contrast, the SOPT, TOL, and FFDI subtests all exert a very high demand on working memory whether directly (SOPT and Digit Span FFDI subtest) or through mental manipulations (which is one of the elements of working memory) that have to be made while the child keeps bead positions (TOL) or numbers (Arithmetic FFDI subtest) in active memory. Further work will be needed to confirm this tentative hypothesis.

While Barkley et al. (see introduction) [24] found mostly psychopathological differences between the ADHD genotype groups, we evidenced a difference in executive function performance. This being said, in the only task that overlaps between our study and theirs (ie WCST), no difference was found between groups. The other two cognitive tasks that Barkley et al. administered and that may be construed as measures of executive functioning were a Continuous Performance Task and the Matching Familiar Figures Test. None of these two tasks yielded a significant genotype main effect. The fact that these two tests also exert rather low demands on working memory may further be viewed as supporting the above-mentioned working memory hypothesis.

As stated in the introduction and in apparent contradiction with the current findings, Cornish et al. [22] and Loo et al[17] have reported a relatively poor performance for carriers of the 10/10 DAT1 genotype. However, Cornish et al. conducted their work on a sample of community controls and not on ADHD children while Loo et al. have compared, in ADHD children, those with the 10/10 DAT1 genotype to a compound group where children with the 9/9 or 9/10 genotypes were pooled together [17]. In a more recent work, Mill et al reported that IQ is lower for those with a combination of putative genetic risk in DRD4 (7-repeat allele) and in the dopamine transporter (10/10 genotype) genes [18]. The combination of genotypes in two separate loci and the use of two different genetic models in each of these loci preclude us from comparing our results with this latter study.

As the10-repeat allele has frequently been reported as being a risk allele for ADHD, it may appear surprising that those with the 10/10 genotype are not the ones with the worst performance here. However, whether the 10-repeat allele is or not a risk allele for ADHD is not necessarily, by itself, very informative about its potential association with a specific endophenotype 4(e.g. executive functioning) for a subset of subjects with ADHD as a given.

While the current study has strengths, including a restricted age range and the assessment of executive function using multiple indices while children were not taking psychostimulant medication, its results should be interpreted with important limitations kept in mind. First, it is noteworthy that some children were tested on the WISC-III by different examiners prior to the initiation of the study. However, if this had an impact on results, it is expected to have manifested itself as statistical noise, making results less likely to appear as significant. Second, the study sample was mainly comprised of children with the combined type of ADHD and not enough subjects had the inattentive or hyperactive/impulsive subtypes in both genotype groups, precluding us from analyzing the relation between DAT1 gene genotype and executive function in each ADHD subtype. Further investigation in larger groups would allow analysis of the aggregation of different clinical aspects stratified by genotype. Third, the polymorphism that we studied in this sample is located in the 3'-untranslated region of the *SLC6A3 *gene and thus does not change the structure of the DAT1 protein. Nonetheless, it has been suggested that it may affect the level of expression of the DAT1 gene, resulting in variable dopamine transporter phenotypes. While this may be the case, the issue still remains controversial. Indeed, it has been reported that DAT binding availability is significantly lower for subjects who are homozygous for the *SLC6A3 *VNTR 10-repeat allele compared to carriers of at least one 9-repeat allele [53] while the opposite association [54] was reported in another study and yet no association [55] was found in a third.

## Conclusion

In conclusion, results from this study show that the DAT1 gene genotype can be a marker of performance of executive functioning (perhaps mostly working memory) in children with ADHD with those having the 9/10 genotype generally exhibiting a relatively poorer performance than those with the 10/10 genotype. As such, and assuming that an endophenotype has a greater potential to target one of the many pathophysiological deficits that in combination may lead to a disorder, present results support the view that it may be possible to characterize with a certain degree of refinement the associations between genotype and semiology. In the wake of frequent false positive findings in behavioral genetics, replication of the current findings is warranted before firm conclusions can be drawn.

## Competing interests

The authors declare that they have no competing interests.

## Authors' contributions

SK analyzed results, conceived, and wrote the manuscript. NG helped design the study, assessed the patients, provided conceptual input on the manuscript content, and provided access to patients. ES-B wrote part of the manuscript and provided conceptual input on the manuscript content. AD provided conceptual input on the manuscript. JB provided conceptual input on the manuscript. VM participated in the cognitive testing of the subjects. AP recruited and cognitively tested the subjects. MT-S recruited subjects and was the coordinator of the project. RDG carried out the molecular genetic studies. JB recruited and cognitively tested the subjects. SS carried out molecular genetic studies and provided conceptual input on the manuscript content. RJ designed the study, assessed the patients, and supervised the conception and writing of the manuscript.

## Pre-publication history

The pre-publication history for this paper can be accessed here:


